# Functional and Resistome Profiling of Paediatric Airway Microbiota in Asthma Using Shotgun Metagenomics

**DOI:** 10.3390/biomedicines14040772

**Published:** 2026-03-28

**Authors:** Aisha Alamri, Abdullah K. Almutairi, Fatimah AlSinan, Ali Alramadhan, Faye Aldehalan, Hatim Almutairi, Mashael Alghuraybi, Norah M. AlHarbi, Shouq F. Alghannam, Sarah S. Alotaibi, Mohammad AlOmary, Suzan AlKhater

**Affiliations:** 1 Department of Clinical Laboratory Sciences, College of Applied Medical Sciences, Imam Abdulrahamn Bin Faisal University, Dammam 34212, Saudi Arabia; faaldehalan@iau.edu.sa; 2 Department of Pediatrics, College of Medicine, Imam Abdulrahman Bin Faisal University, Dammam 34212, Saudi Arabia; akalmutairi@iau.edu.sa; 3 King Fahad University Hospital, Al-Khobar 34445, Saudi Arabia; 4 Pediatric Pulmonary Unit, Qatif Central Hospital, Eastern Health Cluster, Qatif 32654, Saudi Arabia; fhalsinan@moh.gov.sa (F.A.); aalramadhan@moh.gov.sa (A.A.); 5 National Livestock and Fisheries Development Program (NLFDP), Riyadh 13413, Saudi Arabia; hatim.almutairi@hotmail.com (H.A.); ms.alghuraybi@gmail.com (M.A.); norahmoh.alharbi@gmail.com (N.M.A.); 6 Applied Genomic Technologies Institute, King Abdulaziz City for Science and Technology (KACST), Riyadh 11442, Saudi Arabia; shouq.alghnnam@gmail.com (S.F.A.); sarah.sultanotb@gmail.com (S.S.A.); malomary@kacst.gov.sa (M.A.)

**Keywords:** paediatric asthma, airway microbiota, antibiotic resistance gene, shotgun metagenomics, resistome, virulence factor

## Abstract

**Background/Objectives:** Asthma affects millions of patients worldwide and impacts their quality of life, particularly among children. Colonisation or an imbalance within natural resident microbiota may drive inflammatory responses in asthma; antibiotic resistance genes (ARGs) have also been investigated in asthma microbiome studies. However, research on the association between airway microbiota and ARGs remains limited. Therefore, we elucidated functional-level characterisation at the level of ARGs, virulence factors, and active pathways among a paediatric asthma cohort relative to a healthy control. **Methods:** Overall, 29 children with asthma and 20 control subjects were enrolled, and 3 swabs (2 nasal and 1 oropharyngeal) were obtained from each participant. Genomic DNA was extracted and sent for shotgun sequencing, after which bioinformatic analysis was conducted to remove human reads and analyse the microbiota pattern in the samples. The abundance of antibiotic resistance genes was evaluated along with the distribution of virulence genetic markers. Functional investigation of the most prevalent metabolic pathways was also performed. **Results:** Upper airway microbiome functional capacity varied by anatomical location, with oropharyngeal communities exhibiting greater metabolic breadth than nasal communities, suggesting the sample source to be the dominant factor shaping gene content, pathway profiles, and community structure. Asthma-related functional differences were modest, and no biological pathways remained significant following false discovery rate correction. Enrichment of antimicrobial resistance genes was observed, particularly those conferring resistance to β-lactams, macrolides, and tetracyclines. **Conclusions:** Different anatomical niches exhibit differential activities, and further exploration in this direction could aid in the development of diagnostic and therapeutic biomarkers for asthma.

## 1. Introduction

Asthma is a common disease with a rapidly increasing global prevalence [1] and is characterised by hyperresponsiveness of the airways and symptoms such as cough, wheezing, and chest tightness. It exhibits a heterogeneous nature and is driven by complex interactions between genetic predisposition and environmental factors [2].

Over the past decade, the respiratory microbiome has gained increasing attention due to its critical role in defining asthma initiation and progression [3]. Such microbiomes comprise a variety of genomic signatures of archaeal, bacterial, viral, and fungal communities that interact with one another and with immunological mediators, forming a diverse and unique ecosystem that protects the airways [4]. Additionally, advancements in sequencing technology and improvements in bioinformatics analysis tools have enabled more in-depth insights into how this pool of microorganisms interacts which manifests as functional and metabolic pathways and, more importantly, the antibiotic resistance gene clusters [5].

Studies focusing on microbiome development during the first 3 years of life have reported significant fluctuations in microbial diversity and abundance and that these changes are markedly affected by the childbirth route, exposure to infections, antibiotic treatment, and allergies [6]. In the context of asthma, certain bacterial colonisers, including *Hemophilus* spp., *Veillonella*, *Prevotella*, *Moraxella*, *Staphylococcus*, and *Streptococcus*, appear to play a major role in disease development based on data collected from asthmatic airway samples (e.g., nose, nasal wash, and throat) [7].

Notably, these organisms remain a part of the core microbiome in healthy airways; however, the diversity and abundance measures vary across different cohorts, suggesting that colonisation or an imbalance within natural resident microbiota drive inflammatory responses in asthma [3].

The fungal fingerprint has also been evaluated with asthma as a model disease in multiple reports [8]. Certain fungal genera, such as *Aspergillus*, *Candida*, *Saccharomyces*, and *Mucor*, have been reported to be enriched in asthmatic airways and correlated with disease severity [8].

Antibiotic resistance gene pools, collectively referred to as the resistome, which represent the overall composition of antibiotic resistance genes (ARGs), have also been investigated in asthma microbiome studies [5]. When ARG burden was estimated in an asthma cohort, enrichment of a subset of genes was noted, including *ermF*, *ermB*, *ermA* (macrolide resistance), *van* (vancomycin resistance), *cfX* (β-lactam resistance), and *tet* (*tetracycline* resistance), along with other drug-resistance markers based on collected stool samples [9]. However, most of the work has focused on stool samples, and research on the association between airway microbiota and ARGs remains limited. Moreover, translational activities and pathways within microbiota communities represent another aspect for exploration, given that microbial communities are dynamic living populations undergoing continuous replication. Therefore, understanding this level may shed light on which pathways are selectively expressed during health or disease [5].

Previous local studies, including our earlier exploratory work using targeted 16S rRNA and ITS sequencing, have primarily focused on taxonomic characterisation of airway microbiota in paediatric asthma [2]. In the present study, we aimed to elucidate functional-level characterisation at the level of ARGs, virulence factors, and active pathways among a paediatric asthma cohort relative to healthy control using in-depth, shot-gun sequencing technology.

## 2. Materials and Methods

### 2.1. Recruitment of Participants

#### 2.1.1. Inclusion Criteria

We enrolled Saudi children, aged 3–15 years, with clinically diagnosed asthma who were attending the Pediatric Pulmonary Diseases clinic at Qatif Central Hospital (QCH), Qatif, Saudi Arabia. Asthma control status was recorded based on the presence or absence of asthma exacerbations (controlled and uncontrolled status) [10]. Healthy controls without asthma or atopy were recruited along with the patients with asthma.

#### 2.1.2. Exclusion Criteria

Children with respiratory conditions resembling asthma (e.g., cystic fibrosis, chronic infections, or pulmonary structural lesions) were excluded to ensure diagnostic accuracy.

### 2.2. Sample Collection and Processing

Sample collection was carried out prospectively between May 2023 and April 2024. From each participant, one oropharyngeal swab and two nasal swabs (right and left) were collected and suspended in DNA/RNA Shield sample collection tube (Zymo Research, Irvine, CA, USA), and stored at −80 °C until further processing. DNA was extracted from each tube using the QiaAMP DNA miniprep kit (Qiagen, Hilden, Germany) following the manufacturer’s recommendations (Supplementary File S1). DNA from both nasal swabs was pooled into a single tube and analyzed as a single sample.

### 2.3. Whole Genome Sequencing Library Preparation

Genomic DNA (450 ng), quantified using a Qubit Fluorometer 2.0 (Thermo Fisher Scientific, Waltham, MA, USA), was processed using bead-linked transposome tagmentation. Libraries were purified, PCR-amplified using dual i5/i7 index adapters (Illumina Inc., San Diego, CA, USA), and size-selected using magnetic beads, yielding an average fragment size of approximately 600 bp. Library quality was assessed using the LabChip^®^ GX II Touch 24 (PerkinElmer, Waltham, MA, USA), normalised to 2.7 nM, denatured according to Illumina protocols, and sequenced on a NovaSeq 6000 system (Illumina Inc., San Diego, CA, USA) using an S2 flow cell (200 cycles) (Supplementary File S1).

### 2.4. Quality Control and Read Pre-Processing

Paired-end Illumina metagenomic reads were evaluated for base quality, adapter content, and overall read integrity using FastQC v0.12.0 [11]. The reports were aggregated using MultiQC v1.28 [12]. Trimming and quality filtering were performed using fastp v0.23.2 [13] in paired-end mode (-i/-I) with automatic adapter detection (--detect_adapter_for_pe) and default quality thresholds. Post-trimming read quality was re-assessed using FastQC and summarised using MultiQC. Human-derived reads were removed by excluding sequences classified as *Homo sapiens* (taxid 9606) using Kraken2 v2.1.3 [14] prior to downstream functional analysis (Supplementary File S1).

### 2.5. Microbial Diversity Analysis

Raw metagenomic reads were classified using Kraken2 v2.1.3, and bacterial (taxID 2) and fungal (taxID 4751) reads were selectively extracted using KrakenTools and subsequently reclassified against the Kraken2 PlusPF database v20250714 [15]. Microbial diversity was assessed from taxon-level read counts. Alpha diversity was estimated using Observed operational taxonomic units (OTUs) and Shannon, Simpson, and Chao1 indices, while beta diversity was estimated using Bray–Curtis dissimilarity with Principal Coordinate Analysis (PCoA) for visualisation. Group differences were tested using PERMANOVA (999 permutations), while alpha-diversity comparisons were performed using the Mann–Whitney U or Kruskal–Wallis test, as appropriate (Supplementary File S1).

### 2.6. Functional Profiling

De novo metagenomic assemblies were generated using metaSPAdes v3.15.5 [16]. In the initial assembly stage, trimmed paired-end FASTQ files from all samples were used as input, and assemblies were performed independently using 300 GB of memory and 40 computational threads per sample. Assembly quality metrics were generated using abyss-fac v2.3.7 from the ABySS toolkit [17] (Supplementary File S1).

#### 2.6.1. Functional Screening of Resistance and Virulence

ARGs were identified from metagenomic assemblies generated using metaSPAdes in NCBI AMRFinderPlus v4.0.23 [18]. Only high-confidence ARG hits with ≥90% reference coverage were retained. For each hit, a quantitative strength score was calculated by combining the proportional coverage and identity values, which were then aggregated at both the gene and antimicrobial class levels per sample. ARG profiles were integrated with taxonomic abundance data generated by Bracken [19], and pairwise Spearman’s rank correlations were computed between microbial taxa and ARGs to assess potential ecological or functional associations. Spearman’s correlation was selected for its robustness to zero inflation and non-normal distributions, which are typical of metagenomic data. Multiple testing correction was applied using the Benjamini–Hochberg false discovery rate (FDR) procedure (Supplementary File S1).

#### 2.6.2. Functional Profiling and Pathway Analysis

Functional profiling of metagenomic samples was performed using HUMAnN v3.9 via biobakery3 [20]. HUMAnN generated gene families, pathway abundances, and pathway coverage profiles, collectively describing the functional potential of the microbial communities. Gene families were identified using translated searches against the UniRef90 database [21] and nucleotide-level mapping against the ChocoPhlAn species-level genome bin (SGB) pan-genome database, which enabled the detection of both well-characterised and poorly annotated taxa. Gene family abundance was reported as reads per kilobase (RPK) and normalised to copies per million (CPM) to account for sequencing depth. All detected UniRef90 gene families were retained for downstream analysis. UniRef90 identifiers were converted to Kyoto Encyclopedia of Genes and Genomes (KEGG) Orthology (KO) terms using the built-in mapping utility of HUMAnN to enable functional aggregation. KO features were summarised as mean CPM values across asthma vs. control groups and across sampling sites (nose vs. oropharynx), and the most divergent KOs were visualised using z-score-scaled heat maps (Supplementary File S1).

#### 2.6.3. Pathway Reconstruction and Differential Analysis

Functional pathways were reconstructed using MetaCyc within HUMAnN, with additional mapping to KEGG pathways for interpretability. MetaCyc was selected owing to its curated pathway definitions and stringent completeness criteria. Prior to differential testing, pathways classified as UNINTEGRATED, duplicated, absent in either group, or consisting entirely of zero values were excluded. Differential pathway abundance between the asthma and control groups was assessed using the Wilcoxon rank-sum test, and Benjamini–Hochberg FDR correction was applied to account for multiple testing (Supplementary File S1).

#### 2.6.4. Functional Diversity Analysis

Functional alpha diversity was calculated from CPM-normalised UniRef90 gene family profiles using the Observed gene families and Shannon and Simpson indices. Differences between clinical and biological groups (disease status, sampling site, sex, and combined categories) were evaluated using Kruskal–Wallis tests, followed by pairwise Mann–Whitney U tests with FDR correction, where appropriate. Functional beta diversity was assessed using square-root-transformed Bray–Curtis dissimilarity, and group-level differences in functional composition were tested using PERMANOVA (999 permutations) implemented in Scikit-Bio (Supplementary File S1).

### 2.7. Statistical Analysis

Alpha diversity metrics (Observed taxa, Shannon, Simpson) were calculated from taxonomic and gene family abundance tables. Between-group comparisons were performed using Mann–Whitney U or Kruskal–Wallis tests as appropriate. Beta diversity was assessed using square-root–transformed Bray–Curtis dissimilarity, visualised by Principal Coordinates Analysis (PCoA), and tested using PERMANOVA with 999 permutations. Differential abundance of antimicrobial resistance genes, KEGG Orthologs, and MetaCyc pathways were evaluated using Wilcoxon rank–sum tests. Spearman correlation was used to assess associations between taxa and resistance genes. Multiple testing correction was applied using the Benjamini–Hochberg false discovery rate (FDR), with significance set at *p* < 0.05.

## 3. Results

### 3.1. Samples

In total, 29 children with asthma (18 male; 11 female) and 20 control individuals (13 male; 7 female) in the age range of 3–14 years were enrolled in the study (a total of 58 swabs were collected from patients and 40 from controls). Within the asthma cohort, almost 70% of the cases were categorised as controlled asthma status. With respect to the medications used to manage asthma, fluticasone-based therapy was the main managing regimen (monotherapy in 48.4% of patients). Combination therapy with inhaled corticosteroid and long-acting β_2_-agonist (fluticasone/salmeterol or budesonide/formoterol) was used in 16.1% of patients. Montelukast was prescribed either as monotherapy (6.5%) or in combination with inhaled corticosteroids (19.4%). Additionally, Salbutamol was provided as rescue therapy for all asthma cases as needed.

### 3.2. Sequencing Data Summary

A total of 98 samples were subject to DNA extraction and shot-gun sequencing, which generated a mean of 29,012,083 raw reads per sample. After trimming and quality filtering, an average of 1,299,115 reads per sample remained for downstream analysis.

### 3.3. Alpha and Beta Diversity Analyses of Bacterial and Fungal Microbiota

Across both bacterial and fungal communities, anatomical site (nose vs. oropharynx) was the strongest determinant of microbial diversity. Oropharyngeal samples consistently showed higher richness than nasal samples. In the bacterial microbiome, Observed OTUs (*p* < 0.0001) and Chao1 (*p* < 0.0001) were significantly higher in oropharyngeal samples, whereas evenness metrics showed no significant differences (Shannon, *p* = 0.1564; Simpson, *p* = 0.596). Fungal richness followed a similar pattern, with significantly higher Observed OTUs in oropharyngeal samples (*p* < 0.0001); however, the Chao1, Shannon, and Simpson indices were not significantly different (*p* > 0.05). In contrast, clinical status (asthma vs. healthy) did not affect alpha diversity in either domain, with all metrics showing non-significant results (bacterial OTUs, *p* = 0.8831; fungal OTUs, *p* = 0.7063; remaining metrics, *p* > 0.17). Sex also showed no significant effect.

Beta diversity analysis revealed distinct clustering of bacterial communities by sampling site (nose vs. oropharynx), highlighting the latter’s dominant influence, whereas no separation was observed based on asthma status or sex. Fungal communities showed no meaningful clustering across groups.

**Figure 1:**
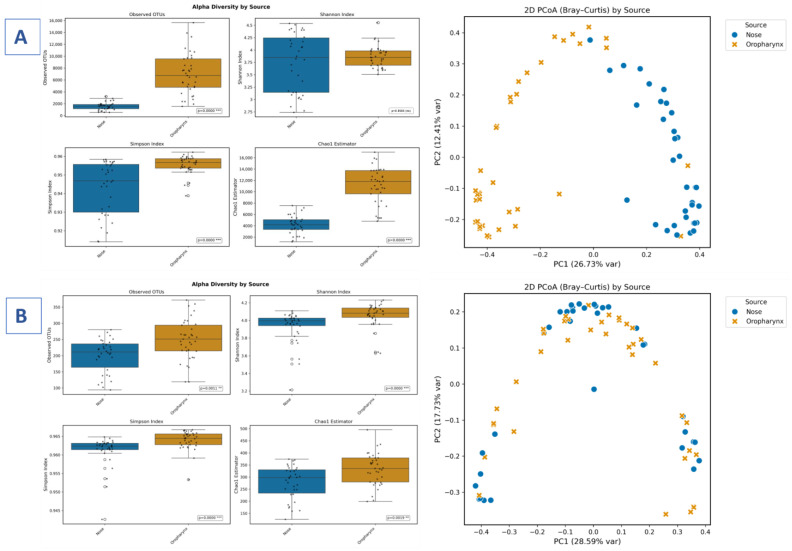
Microbial diversity. (**A**) Bacterial alpha and beta diversity. (**B**) Fungal alpha and beta diversity. Statistical significance is indicated as follows: ** *p* < 0.01; *** *p* < 0.001; ns, not significant.

PERMANOVA confirmed sampling site as the only significant factor affecting bacterial community structure (*p* = 0.001), while asthma status, sex, and all fungal community comparisons remained non-significant (*p* > 0.05) (Figure 1A,B).

### 3.4. Functional Analyses of Metagenomic Antimicrobial Resistance (AMR) of Bacterial Microbiota

**Figure 2:**
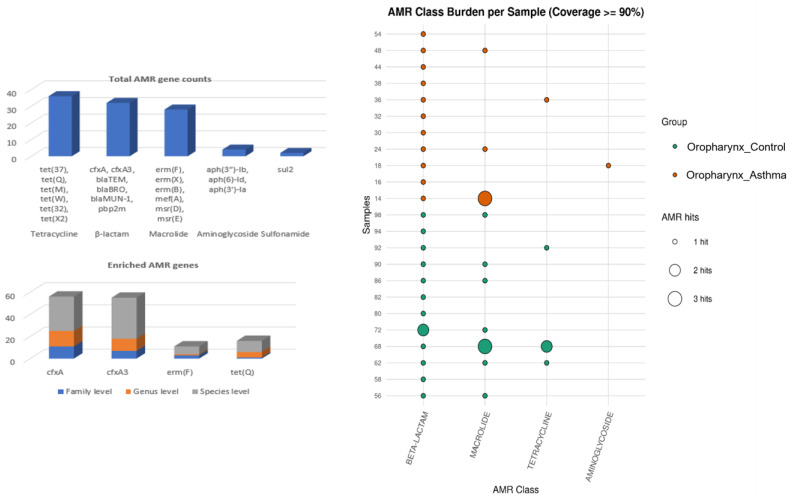
Enriched antimicrobial resistance (AMR) genetic markers across taxonomic ranks.

After filtration, multiple AMR genes were identified. The AMR signal was restricted to oropharyngeal communities. Analysis of ARGs at ≥90% coverage revealed a number of resistant gene classes across the entire cohort, including aminoglycoside (n *=* 4), β-lactam (n = 32), sulfonamide (n *=* 2), tetracycline (n = 36), and macrolide (n = 28) resistance genes. The most frequently detected genes were *cfxA*, *cfxA3*, *erm(F)*, and *tet(Q)* (Figure 2).

Comparison of clinical groups revealed that both the oropharynx_Asthma and oropharynx_Control groups harboured similar ARG class profiles. ARG-microbe association analysis, which was performed to assess whether the enriched resistant markers were associated with the abundance of certain taxa, initially revealed positive and negative correlations at raw *p*-value thresholds; however, none of these associations remained significant after FDR correction (Figure 2).

### 3.5. Virulence Markers of Bacterial Microbiota

Virulence determinants associated with bacterial communities inhabiting the airways were analysed, and 12 virulence markers were identified, most of which were from oropharyngeal samples. Table 1 shows that a majority of virulence marker clusters were detected in the genera *Neisseria*, *Haemophilus*, and *Streptococci,* consistent with the fact that these genera are abundant in airway samples. The identified virulence-associated genes included *pilB*, *lipA/B*, *pilT*, *ctrD*, *hmbR*, *katA*, *recN*, *mntC*, *IpxA/C*, *rfaD*, *yhxB*, *galU*, *lytA*, *orfO*, *gmhA*, and *IpcA*.

### 3.6. Functional Pathway Analysis

Across all samples, the observed gene richness ranged from 167 to 120,390 genes, and this gradient was strongly associated with the sampling site. Oropharyngeal samples exhibited the broadest range of 345–120,390 genes, with a mean richness of 25,707.3 genes, reflecting a considerably higher microbial biomass and functional breadth.

Alpha diversity analysis revealed substantial variability in functional gene richness across samples. Across the datasets, all diversity metrics showed clear differences between nose and oropharyngeal samples, with oropharyngeal samples generally exhibiting higher gene richness and greater diversity. When stratified by disease status (asthma vs. control), consistent differences in alpha diversity were observed between the asthma and control groups across all three measures.

In contrast, nasal samples exhibited markedly lower richness, ranging from 167 to 4848 genes, with an average of 792.6 genes. Shannon diversity showed a similar pattern, ranging from 1.13 to 9.01, with oropharyngeal samples occupying the higher range of Shannon index values and nasal samples showing lower Shannon index values, indicating reduced richness and lower functional evenness in the nasal microbiome. Simpson diversity values also varied substantially (0.25–0.96), with oropharyngeal samples exhibiting consistently higher evenness and nasal samples showing strong dominance of a few abundant gene families.

Overall, these results demonstrate that the functional gene composition of upper airway communities is strongly site-dependent, with oropharyngeal samples harbouring both richer and more even functional profiles than nasal samples. The Chao1 metric was not used for gene-level analysis because the functional analysis was based on sequenced reads, which may introduce rare singleton effects, rendering the analysis of this metric irrelevant [22].

Beta diversity analysis using Bray–Curtis distances further reinforced the strong anatomical signal. PCoA revealed a clear separation of samples by body site along the primary axis of variation, whereas asthma status did not produce evident clustering. PERMANOVA quantified these effects, indicating that the sampling site accounted for the largest proportion of variance (R^2^ = 0.249, *p* = 0.00001). In contrast, asthma status accounted for a relatively small fraction of the variance (R^2^ = 0.025, *p* = 0.0439), while sex showed no significant contribution (R^2^ = 0.013, *p* = 0.2396). No significant interaction was identified between asthma status and sampling site (R^2^ = 0.008, *p* = 0.6236).

Collectively, these results indicate that at the gene level, the overall functional richness of the microbes was largely shaped by the sampling site rather than by disease classification.

### 3.7. Gene- and Pathway-Level Analysis (KEGG)

**Figure 3:**
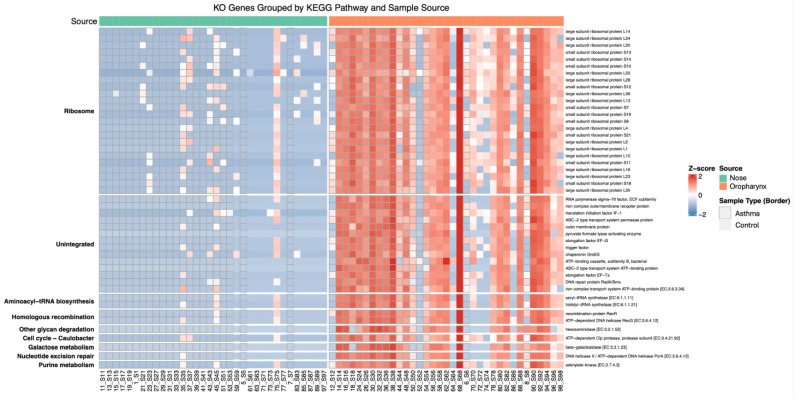
Heatmap showing the 50 KO gene families exhibiting the largest mean CPM differences between samples, grouped according to KEGG pathway annotations. Each row represents a KO gene family ordered by its associated pathway, and each column corresponds to a metagenomic sample. Values are shown as row-wise Z-scores of log10(CPM+1), allowing comparison of relative enrichment patterns across samples. Samples are stratified by Source (nose vs. oropharyngeal, indicated by purple and orange bars, respectively). The heatmap shows that Source (nose vs. oropharynx) is the dominant factor shaping functional gene profiles, with asthma status exerting a weaker effect. CPM, counts per million; KEGG, Kyoto Encyclopedia of Genes and Genomes; KO, KEGG Orthology.

Across the full UniRef90 CPM, 270,690 UniRef90 features were present. Of them, 16,427 features (6.1%) were successfully matched to at least one KO identifier in the mapping database and were therefore retained for KO-based functional profiling. A small subset of UniRef features (76 features; ~0.03%) was mapped to more than 1 KO term, reflecting biological ambiguity (e.g., multidomain proteins or shared sequence homology). These features were proportionally distributed across their assigned KO groups according to HUMAnN’s standard regrouping rules. In both comparisons (asthma vs. control) and (nose vs. oropharynx), the largest expression differences in the KO-level gene families consistently belonged to core ribosomal proteins and a small set of associated translation factors. This indicates that variation in the protein synthesis machinery is a major driver of functional differences among samples (Figure 3).

Across all KO identifiers, the largest CPM differences between the asthma and control samples were mostly associated with housekeeping activities such as ribosomal proteins (K02914; L34, K02874; L14, K02952; S13), sigma-70 transcription factor (K03088), translation initiation factor IF-1 (K02518), and iron complex outer membrane receptor (K02014). The heatmap (Figure 3) shows the relative abundance of certain gene families that reflect microbial metabolic activity. This indicates a strong signal for ribosome-associated Kos; it also suggests that major functional differences among the cohorts are driven largely by translational (ribosomes) pathways rather than by disease-specific pathways, which refer to enhanced bacterial growth activities or taxonomy-associated translational activities. Although the asthma and control groups exhibited differences, anatomical site exerted a stronger influence. Additionally, enrichment of some housekeeping pathways, including aminoacyl-tRNA biosynthesis, DNA replication and repair, cell cycle/homologous recombination, and urine metabolism, was observed, consistent with ribosomal signals.

### 3.8. Pathway Differential Abundance Analysis

**Figure 4:**
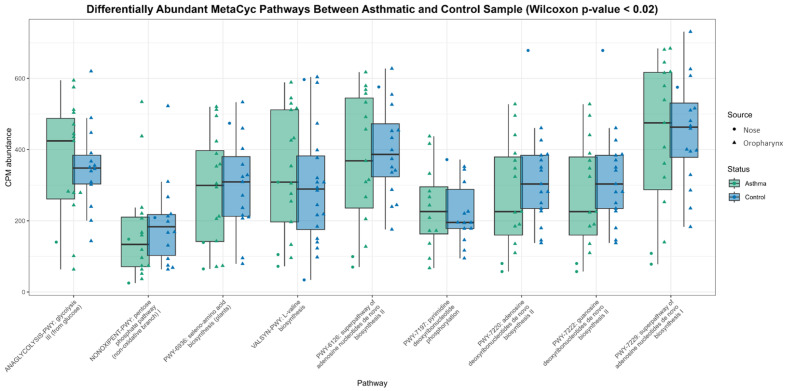
Boxplots showing the relative abundance (CPM) of MetaCyc metabolic pathways that differed between asthmatic and control groups based on Wilcoxon rank-sum testing (*p* < 0.02) for exploratory analysis. Pathway abundances are shown separately for nose and oropharyngeal samples. Results are based on nominal Wilcoxon *p*-values prior to FDR correction.

Although no pathways met FDR-adjusted significance thresholds, several MetaCyc pathways showed raw *p*-values < 0.02 and were retained for visualisation. The differentially abundant pathways included amino acid biosynthesis (e.g., branched-chain amino acid pathways), cofactor metabolism, and nucleotide biosynthesis. These pathways were detected in both groups and exhibited comparable CPM distributions that differed between the asthma and control cohorts at raw *p*-value thresholds. Overall, asthma-related differences in pathway-level abundance were modest; however, several pathways showed consistent shifts in mean CPM between clinical groups, warranting further biological interpretation in the context of microbial functional potential. The set of differentially abundant MetaCyc pathways showed a highly coherent functional pattern, dominated by nucleotide biosynthesis (adenosine, guanosine, and pyrimidine deoxyribonucleotide pathways), folate-dependent one-carbon metabolism, and central carbon metabolism (glycolysis and the pentose phosphate pathway). These pathways were predominantly associated with *Neisseria* and *Streptococcus* (Figure 4).

## 4. Discussion

The nose and oropharynx represent key, exposed, and highly diverse microbial ecosystem within the upper airway, and their microbial composition is closely linked to respiratory tract health and the pathogenesis of asthma.

In the present study, no significant differences were observed between the cases and controls; however, site-specific (nose vs. oropharynx) comparisons revealed robust and significant changes in microbial composition.

Our findings are consistent with those of Broderick et al. [23], who reanalysed raw data from 20 microbiota studies (2624 individuals) on common paediatric respiratory illnesses including asthma. All raw microbiota sequences were re-evaluated using unified bioinformatics tools, and the diversity metrics were re-estimated. Their work revealed that although the disease model tended to exhibit lower microbial diversity and richness, samples collected from patients with asthma did not differ from those collected from healthy controls, suggesting that disease-associated microbiota alterations may be related to functional changes at the taxonomic level rather than changes in microbial richness [23].

Similarly, a recent meta-analysis evaluated published evidence on airway and intestinal microbiota variation in asthma; among the 26 studies included, no consistent differences in microbial alpha diversity between cases and control counterparts were reported while positive differences were observed at the beta diversity levels, indicating a mixed pool of results [24]. Notably, the former study relied mostly on 16s rRNA-generated data and included adult participants with asthma (aged >18 years); therefore, the applicability of the findings to younger populations remains unclear, particularly given the evidence that the microbiome evolves throughout early childhood and tends to stabilise during adolescence (microbiome maturity) [6].

Our functional analysis revealed that several ARGs (resistome) and bacterial virulence markers were predominantly clustered in the oropharyngeal samples and were distributed across both cases and controls. ß-Lactam, macrolide, and tetracycline resistance genetic markers represented the most notable ARGs observed in both cohorts.

Similar findings have been reported by Mac Aogáin et al. [9], who assessed the distribution of airway ARG loads in samples (sputum and swabs from inhaler surfaces) collected from patients with chronic respiratory diseases, including a cohort of patients with severe asthma. Notably, a range of drug-resistance genetic markers (macrolides, β-lactams, fluoroquinolones, and tetracycline) were identified at comparable rates between the case and healthy control groups [9].

A comparable pattern of ARG distribution was described in a longitudinal study investigating the nasopharyngeal microbial-associated resistome of infants over a period of 12 months [25]. Elevated levels of resistance were observed against β-lactams (52%), followed by macrolides (17%) and tetracyclines (12%), and multiple macrolide resistance genes were associated with *Streptococcus*-derived reads [25]; however, the previous study was conducted in healthy participants with an enrichment step for streptococci prior to DNA extraction, whereas our study employed direct shotgun sequencing of airway samples. Notably, concurrent reports have highlighted the presence of an ARG pool in the human microbiome even with no previous antibiotic exposure, raising questions regarding the role of commensals in the dissemination of resistance genes, particularly under selective pressure [26].

In our study, we detected macrolide-encoding markers such as *erm* genes distributed among all study participants. An airway microbiome enriched with macrolide resistance markers (*msrD*, *ermX*, *ermB* and *ermF)* has also been identified in a number of chronic respiratory conditions wherein asthma was studied as a model [9]. The dominant markers of macrolide R genes (*erm*) raise concerns about the utility of the macrolide administration approach in managing respiratory infections [27]. As macrolides (erythromycin and azithromycin) are frequently prescribed to manage respiratory infections, with some studies having demonstrated their utility in preventing Asthma exacerbation episodes [28,29], more studies are needed to explore the enrichment of macrolide resistance among the asthmatic microbiota and to improve prescription practices for patients with asthma.

Of note, the ARG enrichment in the oropharynx may be related to the airway commensal pool, which is known to harbour intrinsic resistance markers, creating a core resistome, rather than specific taxa gene ownership; for example, studies have indicated the presence of *cfx* β-lactam drug markers in *Prevotella* spp. and anaerobic oral microbiota [30,31], suggesting that oral niches are reservoirs of beta-lactamase genes [32]. This is supported by previous reports that identified core ARG and virulence genetic pool following screening of healthy airways, clustered within commensal or potentially non-pathogenic bacteria [33].

Our analysis did not detect differences in ARG enrichment and disease condition, as both cases and controls showed positive signals for ARG presence; this should be interpreted with caution as we did not collect history of antibiotic consumption in both cohorts. This also reflects the persisting gap in knowledge in the field of airway resistome in the asthma population, with the current focus being mostly directed toward elucidating the gut resistome [5]. For instance, Wilson et al. [5] reported enrichment of ARG-Virulence markers in the faecal samples, suggesting co-selection of these genomic traits in the gut of the asthmatic cohort.

Our functional analysis of the microbial pathways reflected the functional stability observed among all individuals enrolled in the study, reflecting the presence of core housekeeping pathways, such as energy production, glycolysis and carbohydrate metabolism, amino acid and nucleotide biosynthesis, and cell wall and envelope biosynthesis. Similar metabolic pathways were identified by Song et al. [34], who assessed functional metagenomic differences among pre-school children with and without wheeze. Although our work could not identify differences at the functional level, other studies have detected significant enrichment of amino acid, fatty acid, and carbohydrate degradation pathways among paediatric individuals with asthma that may be linked to chronic airway inflammation [35].

A majority of functional pathways identified in our cohorts were enriched in *Neisseria* and *Streptococcus*, consistent with the widely recognised notion that these two genera are among the earliest and most persistent colonisers of the airways [36].

The absence of functional differences between cases and controls may not rule out the presence of biologically relevant metabolite changes in the patient samples, warranting further research in this direction [37]. For example, Chiu conducted combined functional pathway analysis of airway samples and metabolite profiling of the sera of individuals with asthma and healthy controls [38]. Their initial functional pathway analysis revealed alterations in metabolic capacity, particularly in the amino acid and lipid pathways, which were mirrored by changes in serum lipid-related metabolite levels [38].

Many factors may be associated with the variability in findings observed across the published microbiota studies, including variations in sampling techniques, utilisation of different sequencing approaches, and heterogeneity among asthma cases. In our study, we used deep shotgun sequencing technology, which is more accurate than the 16s rRNA barcoding approach. Furthermore, stringent FDR corrections were applied to all data to generate findings with less noise (false positive signals) [39].

Asthma heterogeneity, phenotype, endotype, and asthma control level are major factors in shaping microbiota analysis, and mild cases of asthma tend to show comparable microbial fingerprints to healthy controls [40].

Asthmatic cases may acquire other allergies (rhinitis, food, or skin allergies) that manipulate the overall diversity and taxa abundance profiles. Asthma management medications such as bronchodilators and steroids may also affect airway microbiota distribution, making some taxa more prevalent than others, or stressing the microbial pool, allowing certain pathogenic pathways to be expressed more than others [41]. Moreover, geographical location and surrounding environments play major roles in shaping the microbiome enrichment and diversity; a previous study suggested that frequent exposures to enriched microbial environments (e.g., farms) are negatively associated with asthma development [42].

The present study has some limitations, such as the relatively small sample size and the cross-sectional study design, considering that airway microbiota are dynamic communities that actively respond to changes and environmental stimuli. Therefore, the observed findings represent genomic associations that require further functional validation. Evaluating microbial community changes on a longitudinal scale can provide a deeper insight into how these microorganisms interact with each other and how these interactions change in response to inflammation. We used two airway sample types that are known to contain low microbial biomass, making interpretation challenging. Sampling from lower respiratory tract sites (e.g., sputum or bronchoalveolar lavage) may provide a more representative sample of asthma, although the paediatric nature of the cohort renders this challenging. Additionally, data on infection history and antibiotic prescription were missing, limiting the ability to link the resistome profiles with the current status of each participant. As with other microbiome sequencing studies, the generated data may exhibit a degree of annotation bias due to database limitations that favour well-characterised microorganisms, thereby affecting taxonomic profiling and functional analysis accuracy. With ongoing advancements in the field of microbial bioinformatics, higher resolution in data interpretation is expected to emerge in the near future.

Even though asthma control status and medication data were collected from our patients, we were unable to stratify the asthma phenotypes of the patients. Given the heterogeneous nature of asthma, future work should incorporate detailed phenotypic classification with longitudinal sampling to obtain in-depth insights into asthma–microbiome interaction.

## 5. Conclusions

This study examined alterations in the composition and functional activity of microbial communities colonising the airways of children with and without asthma. Although no significant differences were detected at the disease level within the samples tested, marked variation was identified between the two anatomical sites (nose and oropharynx), highlighting anatomical niche as a driver of microbial variation and functional potential. Significant enrichment of ARG markers was also identified in case and control samples; notably, *cfx*, *erm*, and *tet* genes correlated positively with specific airway genera. Of note, the presence of ARGs does not necessarily indicate clinically relevant expression; instead, it likely reflects the airway as a reservoir of ARGs. Whether this ARG pool is linked to previous antibiotic consumption or exposure via other routes (e.g., foods containing antibiotic residues) cannot be determined from the current dataset. Further investigation is warranted, particularly in children with asthma who are frequently exposed to antibiotics, to manage respiratory infections and related exacerbations.

Our study assessed the potential differences between children with asthma and healthy controls at multiple levels using high-throughput shotgun sequencing. Although no significant differences were identified, most previously published studies have relied on targeted approaches (e.g., 16s rRNA and ITS genetic marker sequencing) highlighting the added value of shotgun-based profiling.

Overall, our findings indicate that the identification of asthma-associated biomarkers such as those related to microbiota modulation is necessary to improve paediatric asthma management. Direct metabolite profiling (metabolomic analysis) of patient samples may reveal functional attributes not captured by our functional gene analysis. In addition, although a high-throughput sequencing assay was used in this study, integrating a multi-omics approach that allows for DNA-level analysis along with the analysis of expression levels (RNA transcriptomics) and metabolomics could provide a better understanding of the microbiota-driven metabolic landscape and ultimately improve biomarker discovery for personalised asthma management.

## Figures and Tables

**Table 1:** Virulence-associated genes detected across samples. Species associations were retrieved from VFDB for annotation purposes and not correlated with the current dataset.

Gene	Frequency (Out of 12 Patients)	Function	Common Species (VFDB)
*pilB*	9	Type IV pilus assembly (ATPase)	*Neisseria meningitidis*, *N. gonorrhoeae*, *E. coli*
*pilB*	9	Adhesion and motility	*Neisseria* spp., *Pseudomonas aeruginosa*
*lipB*	8	Lipoic acid transfer (energy metabolism)	*Neisseria* spp., *E. coli*
*pilT*	8	Pilus retraction; twitching motility	*Neisseria*, *Pseudomonas*, *Acinetobacter*
*lipA*	8	Lipoic acid synthesis	*Neisseria* spp., *E. coli*
*ctrD*	8	Capsule export	*Neisseria meningitidis*
*hmbR*	8	Iron acquisition (haemoglobin receptor)	*Neisseria meningitidis*
*katA*	7	Oxidative stress defence (catalase)	*N. meningitidis*, *Staphylococcus aureus*, *E. coli*
*recN*	7	DNA repair	*E. coli*, *Neisseria* spp.
*mntC*	5	Manganese uptake; stress resistance	*Staphylococcus aureus*, *Neisseria* spp.
*lpxA*	3	Lipid A biosynthesis	*Neisseria* spp., *E. coli*
*rfaD*	2	LPS core biosynthesis	*E. coli*, *Salmonella*, *Neisseria*
*yhxB/manB*	2	Cell wall polysaccharide synthesis	*E. coli*, *Neisseria*
*galU*	2	Capsule/LPS precursor synthesis	*E. coli*, *Neisseria*

## Data Availability

The datasets generated and/or analysed in the current study are available from the corresponding author upon reasonable request.

## Supplementary Materials

The following supporting information can be downloaded at: https://www.mdpi.com/article/10.3390/biomedicines14040772/s1.

## Abbreviations

The following abbreviations are used in this manuscript:

AMR	Antimicrobial resistance
KEGG	Kyoto Encyclopedia of Genes and Genomes
ARG	Antibiotic resistance gene
CPM	counts per million
