# Supplementary material for: Functional and Resistome Profiling of Paediatric Airway Microbiota in Asthma Using Shotgun Metagenomics
Source: Biomedicines. 2026 Mar 28;14(4):772. doi: 10.3390/biomedicines14040772 (PMC13113361; doi:10.3390/biomedicines14040772)
Supplement: Supplementary file 1 [file biomedicines-14-00772-s001.zip › biomedicines-4168676-supplementary.pdf]

Functional and Resistome Profiling of Paediatric Airway Microbiota in Asthma Using Shotgun Metagenomics

Methodology Section

This report presents a complete metagenomic analysis workflow including, DNA extraction, shot-gun sequencing protocol, data processing, microbial community profiling, assembly-based characterization, antimicrobial resistance detection and functional analyses.

DNA Extraction

DNA extraction was performed using the QIAamp DNA Mini Kit according to the manufacturer's instructions. Briefly, samples were lysed using proteinase K and lysis buffer to release nucleic acids. Ethanol was then added to facilitate DNA binding to the silica membrane of the spin column. The lysate was transferred to the column and centrifuged to allow DNA adsorption. Contaminants were removed through sequential wash steps using provided wash buffers. Finally, purified DNA was eluted in nuclease-free water or elution buffer and stored for downstream molecular analyses.

Whole Genome Sequencing Library Preparation using Illumina DNA Prep kit - NovaSeq 6000

Whole Genome Sequencing Library Preparation

Whole genome sequencing (WGS) libraries were prepared using the Illumina DNA Prep kit (REF. 1000000025416 v09) and sequenced on the NovaSeq 6000 platform. The DNA concentration was first measured using a Qubit Fluorometer 2.0 to ensure accurate quantification. A total of 450 ng of genomic DNA was used per sample. gDNA was then fragmented using Tagmentation Buffer 1 (TB1) and tagged with adapter sequences using Bead-Linked Transposomes (BLT), followed by reaction termination with Tagment Stop Buffer (TSB). The samples were purified using Tagment Wash Buffer (TWB) to remove impurities. Library amplification was performed using PCR with Enhanced PCR Mix (EPM), incorporating i5 and i7 Index Adapters to ensure compatibility with low plexity pooling and maintain color balance. The amplified products were purified using Sample Purification Beads (SPB) and washed with freshly prepared 80% ethanol. The libraries were then resuspended in Resuspension Buffer (RSB). Library quality assessment was conducted using LabChip® GX II Touch 24, showing an average fragment size of 600 base pairs.

Sequencing Preparation and Sample Run on NovaSeq 6000

The prepared libraries were normalized to a final concentration of 2.7 nM to ensure uniform sequencing conditions. Denaturation was carried out using 0.2 N sodium hydroxide (NaOH), followed by neutralization with 400 mM Tris-HCl, as per the Illumina Denaturation and Neutralization Protocol. The libraries were then loaded onto an S2 Flow Cell (200 Cycles) and sequenced on the NovaSeq 6000 platform according to the manufacturer's specifications.

Bioinformatics analysis

Quality Control and Read Preprocessing

Paired-end illumina metagenomics samples were assessed using FastQC v0.12.0 (Andrews, 2010) to evaluate base quality, adapter content, and overall read integrity. Reports were aggregated using MultiQC v1.28 (Ewels et al., 2016). Trimming and quality filtering were performed with fastp v0.23.2 (Chen et al., 2018) in paired-end mode (-i/-I), with automatic adapter detection (--detect_adapter_for_pe) and default quality thresholds. Post-trimming quality was re-assessed using FastQC and summarized again with MultiQC. In addition, Human-derived reads were removed by excluding sequences classified as *Homo sapiens* (taxid 9606) using Kraken2 v2.1.3 (Wood et al., 2019) for functional analysis.

FastQC analysis revealed high base quality across most reads, though many exhibited 3′ end degradation and adapter contamination. After fastp trimming, read quality improved, and MultiQC summaries confirmed effective adapter removal.

Taxonomic Classification

Raw metagenomic reads were first processed using Kraken2 v2.1.3 to identify and extract reads assigned specifically to bacteria (taxID 2) and fungi (taxID 4751). Read extraction was performed using the extract_kraken_reads.py script from the KrakenTools suite, which applies Kraken2 classification labels to selectively isolate reads belonging to specified taxonomic groups (Lu et al., 2022). This ensured that downstream profiling focused exclusively on bacterial and fungal reads without requiring a separate host-filtering stage. The extracted FASTQ files were subsequently re-classified with Kraken2 using the comprehensive Kraken2 PlusPF database v20250714, curated for broad metagenomic applications (Index zone by BenLangmead).

Kraken2 performs taxonomic assignment through exact k-mer matching, assigning each read to the lowest common ancestor (LCA) of all genomes sharing its k-mer set, enabling rapid and sensitive classification of complex metagenomes (Wood et al., 2019). Because Kraken2's raw read counts may be biased when closely related taxa share substantial genomic similarity, we applied Bracken v3.0.1, a Bayesian re-estimation algorithm that redistributes reads across taxa based on Kraken2's internal k-mer abundance statistics (Lu et al., 2017). Bracken therefore provides more accurate species- and genus-level abundance estimates. Together, KrakenTools, Kraken2, and Bracken form a robust workflow in which Kraken2 provides sensitive read-level classification, KrakenTools enables domain-specific extraction, and Bracken refines abundance estimates, supporting downstream diversity and comparative microbiome analyses.

Microbial Diversity and Abundance

Characterizing the composition and diversity of microbial communities is essential for understanding the ecological structure of the airway microbiome and its potential role in health and disease. Diversity metrics capture the richness and evenness of microbial communities, while abundance profiles identify taxa that dominate specific anatomical sites or clinical groups. Together, these measures provide a comprehensive view of ecological differences across samples, enabling meaningful comparisons between asthma and healthy individuals and between nose and oropharyngeal microbiota.

Microbial diversity was assessed using alpha and beta diversity metrics derived from bracken reports per taxon-level (Lu et al., 2017). Alpha diversity included Observed OTUs (number of non-zero taxa), the Shannon index, the Simpson index, and the Chao1 richness estimator. Beta diversity was quantified using the Bray–Curtis dissimilarity metric, and pairwise distances were calculated for all samples. Principal Coordinates Analysis (PCoA) was performed on a square-root–transformed Bray–Curtis matrix using scikit-bio to visualize differences in community structure. Group-level variation was tested using PERMANOVA with 999 permutations. For alpha diversity, Mann–Whitney U tests were used for two-group comparisons and Kruskal–Wallis tests for multi-group comparisons.

Metagenomic Assembly and Evaluation

De novo Metagenomic assemblies were generated using metaSPAdes v3.15.5 (Nurk et al., 2017). In the initial assembly stage, trimmed paired-end FASTQ files were used as input for all samples, and each assembly was executed independently with 300 GB of memory and 40 computational threads.

Assembly quality metrics were generated using abyss-fac v2.3.7 from the ABySS toolkit (Jackman et al., 2017)

All contigs.fasta files produced by metaSPAdes—whether assembled from trimmed or raw reads—were included in the assessment. For each sample, abyss-fac was used to extract key assembly statistics, including

the total number of contigs, the number of contigs ≥500 bp, L50, N75, N50, N25, E-size, maximum contig length, and total assembly length. The dataset shows substantial variability in assembly quality, reflecting differences in sample complexity, microbial load, and sequencing depth documented in

Functional Screening of Resistance, Virulence, and Plasmids

Antimicrobial resistance genes (ARGs) were identified using NCBI AMRFinderPlus v4.0.23, a curated tool developed by the National Center for Biotechnology Information (NCBI) for detecting acquired ARGs, resistance-associated point mutations, efflux systems, and select virulence determinants from genomic assemblies (Feldgarden et al., 2019). For each sample, the metagenomic assemblies produced by metaSPAdes were used as input.

AMRFinderPlus was run in nucleotide mode (-n) with the --plus option enabled to include the expanded AMRFinderPlus database, which captures stress response and virulence-associated genes in addition to classical ARGs.

A bubble plot visualization was generated to summarize the distribution of ARGs across samples at ≥90% coverage. This approach enabled clear examination of patterns in AMR gene prevalence across Nose–Asthma, oropharynx–Asthma, Nose–Healthy, and oropharynx –Healthy groups.

ARGs present in at least one sample were then merged with corresponding microbial abundance profiles generated by Bracken. Pairwise Spearman rank correlations were calculated between each microbial taxon and each ARG to explore potential ecological or functional associations. Spearman's method was selected because it is non-parametric and robust to the zero inflation, skewed distributions, and compositional nature of metagenomic data. All resulting p-values were adjusted for multiple testing using the Benjamini–Hochberg false discovery rate (FDR) procedure to control for false positives within the large correlation matrix.

Functional Profiling

Gene Level profiling

HUMAnN v3.9 (The HMP Unified Metabolic Analysis Network) was used to perform functional profiling of metagenomic samples (Beghini et al., 2021) through biobakery3 . This pipeline translates raw sequencing reads into biologically functional genes and pathway abundance profiles, enabling the exploration of how microbial communities differ functionally between sample groups. The analysis followed the standard HUMAnN workflow (sequential chart), which generates three key outputs for each sample **(1) a gene families file** reporting the abundance of Uniref90 gene families detects in the sample **(2) a pathway abundance file** summarizing the metabolic pathways present and their estimated abundance, and **(3) a pathway coverage file** indicating the completeness of pathways reconstruction based on detected gene composition. These outputs collectively described the functional potential of the microbial community.

Sequential chart of HUMAnN workflow:

sequenced Reads → Gene level functional profile (nucleotide and proteins) →Pathway level profile (clustering genes to pathways)

The gene families outputs was generated using the Uniref90 protein database for translated search (Uniref90_201901b_full.dmnd) which identifies UniRef90 gene families by aligning quality-filtered reads to the UniRef database and estimating gene abundance using its tiered alignment strategy (Suzek et al., 2015). Nucleotide level functional analysis was performed using the ChocoPhlAn SGB pangenome database (mpa_vJun23_CHOCOPhlAnSGB_202307) This database organizes microbial genomes into species-level genome bins (SGBs), which represent species-level clusters of reference genomes and metagenome-assembled genomes. The SGB system allows functional profiling to capture both well-characterized species and taxa lacking formal names.

Gene family abundances were reported by reads per kilobase (RPK) and were normalized to copies per million (CPM) using HUMAnN's renormalization utility to enable comparison across samples that differ in sequencing depth. All detected UniRef90 gene families including UNMAPPED and UNGROUPED features were retained for gene-level alpha and beta diversity analysis.

UniRef90 gene families quantified by HUMAnN were converted to KEGG Orthology (KO) identifiers using HUMAnN's built-in UniRef-to-KO mapping utility. This step enables aggregation of gene-level abundances into biologically interpretable functional categories. The KO results were filtered to retain only identified KEGG Orthologs entries by removing features labeled UNMAPPED, UNGROUPED. For each KO, mean CPM values were computed separately for asthma and control samples, and for samples obtained from the nose and oropharynx. Features (genes) were ranked by the absolute difference in group means. The top 50 most divergent KOs were selected for heatmap visualization. Abundances were transformed using log10(CPM + 1) and subsequently standardized by z-score scaling (standardization score) across samples.

Pathway level analysis

Gene family profiles were hierarchically clustered into functional pathways using HUMAnN's MetaCyc reaction database (metacyc_reactions_level4ec_only.uniref.bz2) and metacyc pathways version 24 subreactions database. To enable gene-to-pathway interpretation, KO genes described above were also clustered into pathways (KEGG pathways).

Alpha diversity metrics were calculated from the gene family abundance outputs generated by HUMAnN. For each sample four diversity measures were computed: Observed gene families, Shannon diversity, Simpson diversity. All metrics were calculated using a custom python script. To evaluate whether alpha diversity differed between clinical or biological groups, samples were stratified by disease status (Asthma vs Control), sampling site (Nose vs oropharynx), Gender, and a combined category (disease status and Source). Overall group differences were tested using the Kruskal–Wallis test. When more than two groups were present, pairwise comparisons were performed using the Mann–Whitney U test, with Benjamini–Hochberg (BH) correction applied to adjust for multiple testing.

Beta diversity was assessed to characterize differences in functional gene composition across samples. Analyses were performed using the square-root transformed Bray–Curtis dissimilarity, computed from the

CPM-normalized UniRef90 gene family matrix. To evaluate the contribution of clinical and anatomical variables to overall community structure, we performed PERMANOVA (Permutational Multivariate Analysis of Variance) using the permanova function in scikit-bio with 999 permutations. Method validated by (Wilson et al., 2024) The analysis tested the effect of asthma status, sampling site (nose vs. oropharynx), and gender as independent variables. Interaction terms (asthma × sampling site) were also evaluated to determine whether asthma modified the site-specific composition of the functional gene list. All PERMANOVA tests were conducted based on permutation-derived p-values.

Alpha diversity analysis revealed substantial variability in functional gene richness across samples. Across the datasets, all three-diversity metrics showed clear differences between the nose and the oropharyngeal samples, with oropharyngeal samples generally exhibiting higher gene richness and diversity. When stratified by the disease status (asthma and control), consistent differences in alpha diversity were observed between the asthmatic and the control group across all three measures. This indicated that at the gene level, overall functional richness of the microbes was largely shaped by sampling site rather than disease classification.

Choa1 metric was not performed for the gene-level analysis, given that the functional analysis was based on sequenced reads that might show rare singleton effect making the analysis irrelevant (Cameron et al., 2021)

Genes and pathways level analysis: KEGG

To enable functional gene-level analysis, all UniRef90 gene families from the merged CPM matrix were converted into their corresponding KEGG Ortholog (KO) identifiers using the UniRef90→KO mapping file. This conversion provides a standardized functional representation across samples and allows downstream grouping into KEGG pathways.
The conversion process therefore reduced the feature space from the full UniRef90 catalog to the subset of gene families with experimentally supported KEGG annotations, enabling consistent pathway-level interpretation while retaining all functionally annotated information available for this dataset.

Pathway Differential Abundance Analysis

Functional pathway reconstruction was performed using HUMAnN, which maps UniRef90 gene families to MetaCyc reactions and infers pathway presence and abundance based on gene coverage and completeness. MetaCyc was selected as the primary pathway framework because it provides high-resolution, curated biochemical pathway definitions with strict rules for pathway completeness, enabling more precise functional interpretation of metagenomic data.
Before differential analysis, all UNINTEGRATED pathways, duplicated pathway entries, pathways consisting entirely of zeros, and pathways absent from either group (asthma or control) were removed. This ensured that only biologically interpretable and comparable pathways were tested. All pathway abundances were expressed as CPM-normalized values.
Differential abundance between asthmatic and control samples was assessed using the Wilcoxon rank–sum test, applied to each MetaCyc pathway independently. To control for false positives due to multiple hypothesis

testing, False Discovery Rate (FDR) correction was applied across all pathways. No pathways reached significance after FDR correction ($q < 0.05$). However, for exploratory visualization and interpretation, pathways with raw p-values < 0.02, non-zero CPM values, and presence in both clinical groups were retained. Method validated by (Wilson et al., 2024)

The analysis examined pathway differences across multiple biological factors:

- Asthma status (Asthma vs. Control)
- Sample source (Nose vs. oropharynx)
- Gender
- Overall cohort mean abundance patterns

Mean CPM values per group were calculated prior to statistical testing to summarize central tendencies.

References

1. Andrews, S. FastQC: A Quality Control Tool for High Throughput Sequence Data. 2010. Available online: http://www.bioinformatics.babraham.ac.uk/projects/fastqc/ (accessed on 1 May 2025).
2. Ewels, P.; Magnusson, M.; Lundin, S.; Käller, M. MultiQC: Summarize analysis results for multiple tools and samples in a single report. *Bioinformatics* **2016**, *32*, 3047–3048. https://doi.org/10.1093/bioinformatics/btw354.
3. Chen, S.; Zhou, Y.; Chen, Y.; Gu, J. fastp: An ultra-fast all-in-one FASTQ preprocessor. *Bioinformatics* **2018**, *34*, i884–i890. https://doi.org/10.1093/bioinformatics/bty560.
4. Wood, D.E.; Lu, J.; Langmead, B. Improved metagenomic analysis with Kraken 2. *Genome Biol.* **2019**, *20*, 257. https://doi.org/10.1186/s13059-019-1891-0.
5. Lu, J.; Rincon, N.; Wood, D.E.; Breitwieser, F.P.; Pockrandt, C.; Langmead, B.; Salzberg, S.L.; Steinegger, M. Metagenome analysis using the Kraken software suite. *Nat. Protoc.* **2022**, *17*, 2815–2839. https://doi.org/10.1038/s41596-022-00738-y.
6. Nurk, S.; Meleshko, D.; Korobeynikov, A.; Pevzner, P.A. metaSPAdes: A new versatile metagenomic assembler. *Genome Res.* **2017**, *27*, 824–834. https://doi.org/10.1101/gr.213959.116.
7. Jackman, S.D.; Vandervalk, B.P.; Mohamadi, H.; Chu, J.; Yeo, S.; Hammond, S.A.; Jahesh, G.; Khan, H.; Coombe, L.; Warren, R.L.; et al. ABySS 2.0: Resource-efficient assembly of large genomes using a Bloom filter. *Genome Res.* **2017**, *27*, 768–777. https://doi.org/10.1101/gr.214346.116.
8. Feldgarden, M.; Brover, V.; Gonzalez-Escalona, N.; Frye, J.G.; Haendiges, J.; Haft, D.H.; Hoffmann, M.; Pettengill, J.B.; Prasad, A.B.; Tillman, G.E.; et al. AMRFinderPlus and the Reference Gene Catalog facilitate examination of the genomic links among antimicrobial resistance, stress response, and virulence. *Sci. Rep.* **2021**, *11*, 12728. https://doi.org/10.1038/s41598-021-91456-0.
9. J. Lu, F. P. Breitwieser, P. Thielen, and S. L. Salzberg, 'Bracken: estimating species abundance in metagenomics data', *PeerJ Computer Science*, vol. 3, p. e104, Jan. 2017, doi: 10.7717/peerj-cs.104.
10. Beghini, F.; McIver, L.J.; Blanco-Míguez, A.; Dubois, L.; Asnicar, F.; Maharjan, S.; Mailyan, A.; Manghi, P.; Scholz, M.; Thomas, A.M.; et al. Integrating taxonomic, functional, and strain-level profiling of diverse microbial communities with bioBakery 3. *eLife* **2021**, *10*, e65088. https://doi.org/10.7554/eLife.65088.

11. B. E. Suzek, Y. Wang, H. Huang, P. B. McGarvey, C. H. Wu, and the UniProt Consortium, 'UniRef clusters: a comprehensive and scalable alternative for improving sequence similarity searches', *Bioinformatics*, vol. 31, no. 6, pp. 926–932, Mar. 2015, doi: 10.1093/bioinformatics/btu739.
12. Wilson, N.G.; Hernandez-Leyva, A.; Schwartz, D.J.; Bacharier, L.B.; Kau, A.L. The gut metagenome harbors metabolic and antibiotic resistance signatures of moderate-to-severe asthma. *FEMS Microbes* **2024**, *5*, xtae010. https://doi.org/10.1093/femsmc/xtae010.
13. Cameron, E.S.; Schmidt, P.J.; Tremblay, B.J.-M.; Emelko, M.B.; Müller, K.M. Enhancing diversity analysis by repeatedly rarefying next generation sequencing data describing microbial communities. *Sci. Rep.* **2021**, *11*, 22302. https://doi.org/10.1038/s41598-021-01636-1.
